# Frequent Insect Visitors Are Not Always Pollen Carriers in Hybrid Carrot Pollination

**DOI:** 10.3390/insects9020061

**Published:** 2018-06-07

**Authors:** Ann Gaffney, Björn Bohman, Stephen R. Quarrell, Philip H. Brown, Geoff R. Allen

**Affiliations:** 1 Tasmanian Institute of Agriculture, University of Tasmania, Churchill Ave, Hobart 7005, Australia; ann.gaffney@uni.sydney.edu.au (A.G.); stephen.quarrell@utas.edu.au (S.R.Q.); p.h.brown@cqu.edu.au (P.H.B.); geoff.allen@utas.edu.au (G.R.A.); 2 School of Molecular Sciences, The University of Western Australia, 35 Stirling Hwy, Perth 6009, Australia; 3 School of Health, Medical and Applied Sciences, Central Queensland University, Callemondah 4701, Australia

**Keywords:** carrot, *Daucus carota*, hybrid, crop pollination, seeds, pollen transfer, honey bee, *Apis mellifera*, native pollinators

## Abstract

Insect crop visitations do not necessarily translate to carriage or transfer of pollen. To evaluate the potential of the various insects visiting hybrid carrot flowers to facilitate pollen transfer, this study examines insect visitation rates to hybrid carrot seed crops in relation to weather, time of day and season, pollen carrying capacity, inter-row movement, and visitation frequency to male-fertile and male-sterile umbels. The highest pollen loads were carried by nectar scarabs, honey bees, and the hover fly *Eristalis tenax* (Linnaeus). Honey bees and muscoid flies were observed to forage mostly within the male fertile carrot row while nectar scarabs and *E. tenax* foraged across rows, carrying equal pollen loads regardless of their distance from the pollen source. All observed insect taxa were more frequently seen visiting male-fertile than male-sterile umbels. In contrast to other visiting insects, honey bees were abundant and frequent visitors and were observed carrying high pollen loads. Consequently, we suggest both optimizing honey bee management and improving the attraction of carrot lines to honey bees to improve pollination rates for hybrid carrot seed crops.

## 1. Introduction

Effective pollination of carrot seed crops through insect vectors is critical to commercial success. Although surveys of carrot seed crops have revealed that many potential pollinator species visit carrot seed crops at flowering [1,2], poor pollination is a known problem for the growers [3].

For carrot flowers, the duration of flowering in a single crop is approximately six weeks [4], over which time the crop may experience fluctuations in pollinator visitation. During the flowering period, carrot umbels reach receptivity sequentially, starting with the primary umbel and progressing to secondary, tertiary, and if present, quaternary umbels. Each umbel is receptive to pollen for a period of two to four days [5]. This means that a single plant within a crop is potentially attractive to insect pollinators for two to three weeks.

Currently, hybrid carrot seed production is generally reliant on hives of the European honey bee, *Apis mellifera* [6,7]. This strategy has serviced the open-pollinated carrot lines by increasing seed yield and fertility [2]. However, despite single-species pollinator management providing acceptable yields in some seed production systems [8], sole reliance on *A. mellifera* does not always provide adequate yields in hybrid carrot seed crops [9,10]. For this reason, it is being increasingly recognized that species rich wild pollinator assemblages should be utilized to ameliorate the production losses that are observed when relying on a single pollinator species. Indeed, several other insect orders have been demonstrated to provide pollination services to carrot flowers, including Dipteran (Calliphoridae [7], Muscidae [11], Syrphidae and Chloropidae [12]), and Coleopteran species [13]. Furthermore, due to the broad endemic geographic range of wild *Daucus carota*, it has most likely adapted to having pollination services provided by multiple insect orders and/or species [13]. Despite this, *A. mellifera* remains the primary vector for carrot pollen in commercial cropping due to their ease of management compared to wild pollinators [14].

In hybrid carrot seed crops, cytoplasmically sterile (CMS) or male-sterile flower beds up to eight-rows wide are alternated with beds of male-fertile (MF) lines [15], making pollinator movement between rows critical to pollination success. However, *A. mellifera* has been observed to preferentially visit MF plants over CMS field plantings in some studies [4], reducing pollination success of CMS lines with increased distance from MF lines [16,17].

Further compounding this issue is how weather conditions impact insect pollinator flower visitation [18,19,20,21,22,23]. For example, the activity of syrphids, butterflies, and many bee species (including *A. mellifera*) is restricted by cloudy [24,25], cool, windy, humid, or wet weather [26,27]. However, other potential pollinator species including other bee (Megachildae and *Bombus* spp.) and Diptera have demonstrated greater tolerance to these weather conditions [23,28,29]. These findings lend further weight to the argument for maximizing pollinator species richness in agricultural crops to ensure optimal pollination outcomes. This is particularly important when periods of inclement weather overlap with pollination windows [30].

To the best of our knowledge, there have been few published studies of patterns of insect visitation to carrot seed crops [30,31]. Considering the now long-standing debate of the importance of various taxa of insects for crop pollination (see [32] for a recent review), we examined carrot umbel visitation rates, pollen carrying capacity, inter-row movement, and relative visitation frequency to male-fertile and male-sterile (CMS) carrot umbels over a range of insect taxa. Based on our results, we evaluate the efficiency and potential for the various insects as pollinators and highlight possibilities for further research and development of strategies to improve hybrid carrot pollination.

## 2. Materials and Methods

### 2.1. General Background

Carrot seed crops reach maturity throughout summer (December to February in Tasmania). A series of four observation trials (trials 1, 2, 5, 6) and two trapping trials (trials 3, 4) were carried out over three seasons in 2002–2005, at three sites, to monitor insect visitation in flowering carrot crops. Analyses of the data were undertaken to explore relationships between visitation rates with climatic, seasonal, and biological factors. The timing, carrot type, crop area, honey bee hive placement, and insecticide spray history of these field trials is given in Table 1. Commercially sourced hives were present ca. 3 m from all carrot crops in all trials with the exception of trial 1, where no commercially sourced pollination hives were utilized. Full site descriptions and additional management information for each location are available in the supplementary information (Figures S1–S7, Tables S1–S3). The hybrid crops observed represent current management strategies still applied by seed producers.

### 2.2. Weather

Weather data collected at Hobart Airport were provided by the Australian Government Bureau of Meteorology. Hobart Airport Weather Station is located at 42.834° S, 147.503° E, which is 12 km south of the carrot seed crops located at Bejo Seeds Pty. Ltd. and StrathAyr Turf Systems Pty. Ltd. and 6 km southeast of the University Farm.

Weather variables collected were: minimum temperature (°C), maximum temperature (°C), rainfall (mm), bright sunshine (hours), 9:00 a.m. wind speed/direction, and 3:00 p.m. wind speed/direction. Minimum and maximum temperatures were measured at approximately 1.5 m above ground level within a Stevenson Screen. Maximum temperatures were recorded between 9:00 a.m. on the day of the record and 9:00 a.m. on the next day, as was rainfall. Minimum temperatures were recorded in the 24 h prior to 9:00 a.m. on the day of the record. Bright sunshine hours were measured using a Campbell–Stokes recorder. Wind speed and direction were measured at a height of 10 m above the surface. Wind speed was averaged over the 10 minutes leading up to the time of observation. Wind direction was measured using a rotating cup anemometer (Bureau of Meteorology, 2009).

### 2.3. Insect Visitation to Umbels

Observation trials were conducted at three sites within 30 km from Hobart in southern Tasmania, Australia—StrathAyr Turf Systems, Pty. Ltd., Bejo Seeds Pty. Ltd., and the University Farm—over three seasons in four trials: trial 1, 2, 5, and 6 (Table 1). Observations were conducted only on warm (temperature at 9:00 a.m. over 15 °C and maximum temperature over 20 °C), sunny, calm days, confirmed by weather data collected at Hobart Airport Weather Station. Observations were performed by teams of one to three people simultaneously between 9:00 a.m. and 3:00 p.m. At each site, for each observation period, two umbels were randomly selected and observed for 5 min. Umbels were selected across the entire plantings. Umbel selection was done at random but observed pairs were in enough proximity so that an observer could simultaneously observe both at once. Observers were at least 10-m distant from each other and stood at least 1 m from the umbels being observed. Only umbels with an estimated number of receptive flowers >40% were included in the random selection for observations of insect behavior. Prior to each observation period, the number and types of insects already present on the selected umbels were recorded. In trial 6, each observation period was extended to 20 min due to low insect activity.

Each time an insect alighted, it was recorded as belonging to one of 11 groupings: *Apis mellifera*, *Chauliognathus lugubris* (Cantharidae), Coccinellidae, Ichneumonidae, *Phyllotocus* spp. (Scarabaeidae), bees other than *Apis mellifera* (Colletidae, Halictidae, Apidae), other Hymenoptera, other Coleoptera, *Comptosia ocellata* (Bombylidae), *Melangyna/Simosyrphus* spp. (Syrphidae), or muscoid flies. No distinction was made between first-time insect visits and revisitations. For analysis, each umbel was scored separately. To avoid disturbing the insects, each observer remained at least 1 m away and positioned not to cast a shadow over the umbels.

To measure the visiting rate of the 11 insect groupings, observations were repeated across four trials (1, 2, 5, and 6) and three seasons over nonconsecutive days. The timing of the observations, the total number of flowers observed, and the length of each trial varied due to weather conditions (outlined above), the number of flowers available with >40% receptive umbelets, and the number of observers participating in each trial. In trial 1, observations were undertaken over 4 days between 14 December and 24 December 2002, covering 408 umbels; in trial 2, over 10 days between 3 January and 3 February 2003, covering 224 umbels; in trial 5, over 7 days between 15 January and 5 February 2004, covering 1242 umbels; and in trial 6, over 5 days between 30 December 2004 and 13 January 2005, covering 24 umbels. A comparison of the visitation rates between these insect groupings within each field trial was undertaken using Friedman’s test, using each umbel as a block and thereby controlling for differences between umbels. Multiple Wilcoxon signed ranks tests were then used to determine which particular pairs of insect groupings differed from one another. *p* values were adjusted to *p* < 0.001 to preserve 95% confidence when making multiple comparisons of the same data. All statistical analysis was performed using SPSS^®^ version 17.0 (SPSS Inc., Chicago, IL, USA).

To assess timing of visits during the day by insect groupings, a subset of the total insect visitation data from trials 2 and 5 was used for analysis. Each day was divided into three time periods (9:00 a.m. to 11:00 a.m., 11:00 a.m. to 1:00 p.m., and 1:00 p.m. to 3:00 p.m.). In trial 2, observations were undertaken over 8 days between 3 January and 3 February, with 192 umbels observed. In trial 5, observations were undertaken over 5 days between 20 January and 5 February, with 900 umbels observed. To examine whether the visitation frequency to umbels varied for an insect grouping between morning, midday, and afternoon, data for each time period was pooled for each day. This data was subsequently analyzed using Friedman’s test, for 10 of the 11 insect groupings (insufficient numbers of *Melangyna/Symosyrphus* spp. were recorded), using each day as a block, thereby controlling for variation between days. All analysis was conducted using SPSS^®^ version 17.0.

### 2.4. Insect Traps and Analysis of Trap Catches

Static trapping trials were conducted in the second season in a commercial open-pollinated crop at StrathAyr Turf Systems Pty. Ltd. and in an experimental hybrid crop at the University Farm. Sampling was conducted using water traps and yellow sticky traps located within these crops.

All traps were erected just above umbel height (approximately 150 cm above ground level) and were placed in pairs at 2 m apart down the central long axis of each plot. Five sets of trap pairs (one water trap and one sticky trap) were placed at each location, with the traps within each pair placed 30 cm apart. Commercially available 10 × 20 cm double-sided yellow sticky traps (AgriSense^®^ Pontypridd, UK) were rigidly mounted vertically between two sections of pipe on metal star posts. Water traps were utilized using a design modified from [33]. Each trap was made from a yellow plastic container (10 cm deep and 13 cm diameter) and mounted on wooden poles. Each water trap was filled with 600 mL of water containing 10% ethanol as a preservative and a small amount of dish washing liquid (Cusson’s Morning Fresh, Mulgrave, VIC, Australia) to break the surface tension.

Traps were set in season 2 over 59 days (trial 3) and 65 days (trial 4). Sampling in trial 4 began three weeks after sampling in trial 3, providing five weeks overlap. Traps at trial 3 were set between 5 December and 2 February and were cleared 17 times. Traps at trial 4 were set between 27 December and 1 March and were cleared 19 times. Traps were collected twice weekly. New sticky traps were set as used traps were collected while the water traps were refilled. All sticky traps were wrapped in clear cling wrap as they were collected.

All trap catches were examined under a binocular dissecting microscope at 40× magnification. Larger insects (>5 mm) were separated taxonomically as far as possible, resulting in morphological groupings from species level to order level. A subset of the most commonly trapped “larger insects” were chosen for further analysis in relation to weather variables. These were *A. mellifera*, *C. lugubris* (Cantharidae), *Phyllotocus* spp. (Scarabaeidae), Coccinellidae, other Coleoptera, Ichneumonidae, bees other than *A. mellifera*, other Hymenoptera, and muscoid flies.

Presence or absence of the various insect groupings per trap period was analyzed in relation to the following variables: field site, trap type, wind speed at 9:00 a.m., wind speed at 3:00 p.m., wind direction at 9:00 a.m., wind direction at 3:00 p.m., bright sunshine hours, minimum temperature, maximum temperature, and precipitation. Wind speed and direction, bright sunshine hours, precipitation, and minimum and maximum temperatures were averaged over the number of days that each trap was set. Analysis was performed using R© version 2.9.1 (R Foundation for Statistical Computing, Vienna, Austria) using multiple logistic regression. After fitting, the full model terms were dropped based on their significance in the model until the model of best fit was obtained.

### 2.5. Pollen Carrying Capacity, Inter-Row Movement, and Visitation Frequency to Male-Fertile and Male-Sterile Umbels

To examine the pollen carrying capacity of insects found within carrot seed crops, insects foraging on umbels were collected by hand and net from MF and CMS umbels grown at the University Farm during the second season (trial 5). Carrots were planted in single rows and insects were collected from one row of MF carrots (row M) and three rows of CMS carrot umbels (F1, F2, and F4; 0.8 m, 1.6 m, and 3.2 m distant from row M). Only five groupings—*A. mellifera* (honey bees, *n =* 133), *Phyllotocus rufipennis* (nectar scarabs, *n =* 35), muscoid flies (*n =* 243), *E. tenax* (large hoverflies, *n =* 56), and *Melangyna/Symosyrphus* spp. (small hoverflies, *n =* 36)—were collected in sufficient numbers to warrant further examination.

The insects collected were returned to the laboratory and frozen in Eppendorf^®^ centrifuge tubes at −20 °C prior to examination. Before processing for pollen counts, the *A. mellifera* had their hind legs removed to exclude pollen on their corbiculae and enable comparison of pollen on the rest of their body to that of other insects. This also controlled for differences between nectar collecting and pollen collecting bees.

Pollen loads were analyzed using a modified method from [34]. Individual insects were placed in 1.5 mL Eppendorf tubes containing 50 µL of solidified glycerol gelatin (40 g of melted gelatin in 60 mL of glycerol diluted with 100 mL of deionized water). The storage tube from which the insects had been retrieved was flushed with 400 µL of xylene, which was subsequently added to the centrifuge tube containing the insect. A further 400 µL of xylene was added to the centrifuge tube. The tubes were agitated on a vortex mixer for 3 min to displace the pollen from the insect. The insects were then removed and the tubes centrifuged at 15,000 rpm for 1 min, after which the xylene was decanted. The pollen-impregnated glycerol gelatin pellets were removed with a fine-hooked needle, placed on microscope slides, heated to melting, and covered with cover slips. Light pressure was applied to spread an even film of glycerol gelatin over the slide surface beneath the cover slip. After the slides had set they were examined under a light microscope at 100× magnification. Counts of the total number of carrot pollen grains were made from 10 randomly selected fields of view for each slide. The total number of pollen grains collected from each individual insect was calculated from the ratio of the slide area examined in 10 fields of view to the total area of the cover slip. Pollen counts for each insect were sorted into one of four categories (0–9 grains, 10–99 grains, 100–999 grains, and 1000+ grains) and were analyzed using contingency tables between groupings and within groupings using SPSS^®^ version 17.0.

To examine whether each insect grouping visited either MF or CMS umbels more frequently, observations of insect visits to umbels were undertaken in trials 2 and 6. Pairs of MF and CMS umbels were simultaneously observed in both trials. In trial 2, 132 pairs of umbels were observed on 8 days for 5 min per pair, whereas in trial 6, 24 pairs of umbels were observed for 20 min per pair on 5 days. The visitation rates of each grouping to MF and CMS umbels were compared using Wilcoxon signed ranked tests, with each pair being the simultaneous observation of an MF and CMS umbel. Umbel pairs were excluded from the analysis if they had none of that grouping visit during the observation period. All analyses were undertaken using SPSS^®^ 17.0.

## 3. Results

### 3.1. Daily Visitation to Umbels

The numbers of visitors from each insect grouping initially present on the umbels showed a significant correlation to the overall numbers that visited over the subsequent 5-min observation periods (Spearman’s r = 0.770, *p* = 0.009). Therefore, the visitation counts report the combined number of insects present at the start of the count pooled with those visiting during the count interval. Insect groupings initially observed on the umbels agreed with that expected from a random distribution across all umbels with the major exception of both beetle groups (Poisson fit: *Phyllotocus* spp.: χ^2^ = 160.4, soldier beetles; χ^2^ = 111.9, all df = 2 and *p* < 0.001). Both beetle groups were significantly aggregated, with variance to mean ratios (VMRs) much greater than 1: *Phyllotocus* spp. (1.42) and soldier beetles (1.49), although no more than three beetles were present on any one umbel.

The rates of insect visitation for each insect grouping varied greatly between trials and between and within seasons, for example, during trials 1 and 2 in the first season and between years. The most frequent visitors in trial 1 (the only trial which did not have *A. mellifera* hives placed nearby) were *P. rufipennis* at 3.8 visits/5 min. In trial 2, *A. mellifera* was most frequently observed at 6.7 visits/5 min. In the second season, the most frequent visitors in trial 5 were *A. mellifera* and *C. lugubris* at 0.33 visits/5 min and 0.26 visits/5 min, respectively. In the third season, muscoid flies (5.6 visits/20 min), as well as *Phyllotocus* spp. (predominantly *P. macleayi*) (2.7 visits/20 min), *A. mellifera* (3.9 visits/20 min), and *C. lugubris* (3.7 visits/20 min), were all frequent visitors to umbels. *A. mellifera* and muscoid flies appeared to be the most consistent taxa across all four trials, as they were consistently within the first three rankings of the most frequent visiting groupings.

**Figure 1:**
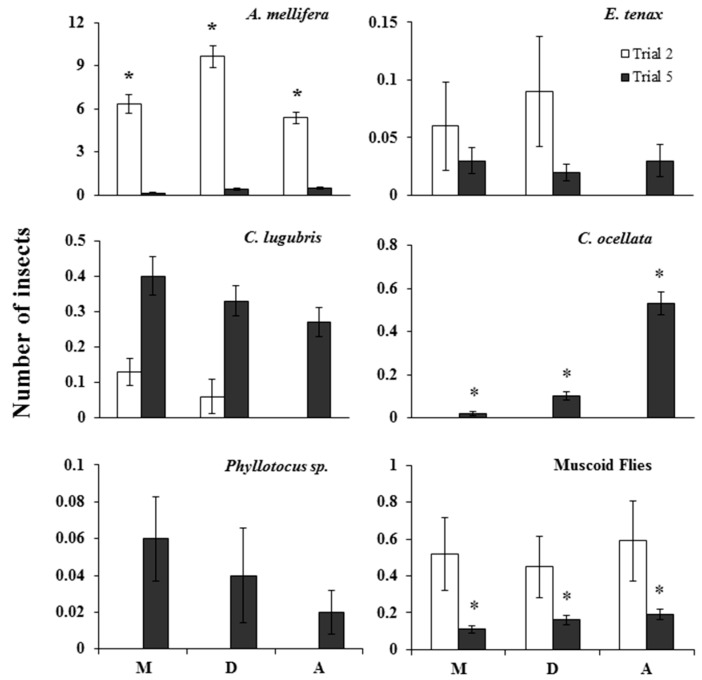
Number of insects (mean ± SEM) observed visiting carrot inflorescences for 5 min in the morning (M), midday (D), and afternoon (A) in trials 2 (*n =* 8 days) and 5 (*n =* 5 days). * Indicates significant differences at *p* < 0.05 using Friedman’s test.

Insect visitation in the morning, midday, and afternoon in the two field trials (2 and 5) showed varying patterns across seasons (Figure 1). In trial 2, *A. mellifera* visited carrot umbels in significantly different numbers across the three time periods (Friedman test: χ^2^ = 12.25, df = 2, *p* = 0.002), with highest visitations in the middle of the day. Numbers of *A. mellifera* were too low in trial 5 for meaningful comparisons, although both *C. ocellata* (χ^2^ = 9.58, df = 2, *p* = 0.008) and muscoid fly (χ^2^ = 7.00, df = 2, *p* = 0.03) visits significantly increased as the day progressed during this trial, while visitations of *C. lugubris* (trial 2 and 5) and *Phyllotocus* spp. (trial 5) decreased as the day progressed. However, the decrease in *Phyllotocus* beetle visitation was not significant. Furthermore, there was no significant correlation between visitations and time of day for coccinellids, wasps, other bees, or the loose grouping of “other insects” (*p* > 0.05).

### 3.2. Insect Activity Over the Field Season

**Figure 2:**
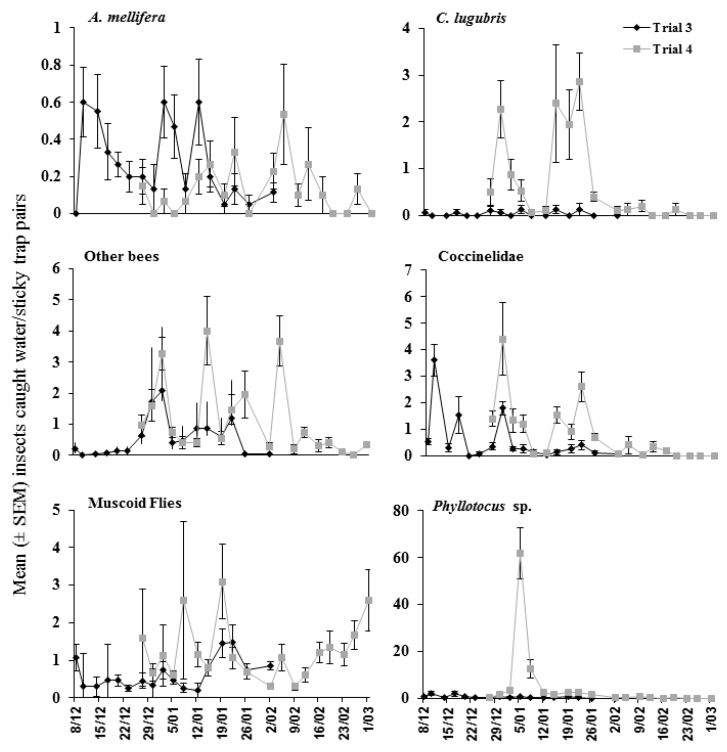
Number of insects (means ± SEM) caught in water traps and sticky traps (data pooled) in trials 3 and 4.

The trap type influenced the likelihood of some species being captured, with greater numbers of *A. mellifera* (Z = −2.693, *p* = 0.007), coccinellid beetles (Z = −3.222, *p* = 0.001), ichneumonid wasps (Z = −3.031, *p* = 0.002), and other bees (Z = −2.142, *p* = 0.032) caught on sticky traps compared to the water traps. Of the insect fauna collected, only *C. lugubris* numbers significantly differed between sites, with a more frequent presence at field site 4 (Z = 3.500, *p* < 0.0005; see Figure 2 for catches of insect groupings over time). *Phyllotocus* beetles (nectar scarabs) were found infrequently with the exception of trial 4, where they were observed over a period of ca. one week in early January (Figure 2).

Maximum temperatures varied widely over the trapping period, ranging from 15 to 36 °C, though only 4 days exceeded 30 °C, whereas minima varied between 4 and 18 °C. However, temperature was only a significant predictor of insect abundance for two insect groups: *A. mellifera*, where increases in daily minimum temperature increased their activity (Z = 2.300, *p* = 0.021), and other beetles (a grouping that excludes *Phyllotocus* spp., coccinellids, and *C. lugubris*) (Z = 2.218, *p* = 0.027), for which trap catches increased with increased daily maximum temperatures.

Wind speeds varied between 0 and 50 km/h at 9:00 a.m. and were typically stronger at 3:00 p.m., ranging between 9 and 48 km/h. Lower wind speeds at 9:00 a.m. were associated with an increased trapping of coccinellid beetles (Z = −2.640, *p* = 0.008) and higher wind speeds at 3:00 p.m. with increased trapping of *C. lugubris* (Z = 2.962, *p* = 0.003) and coccinellid beetles (Z = 2.836, *p* = 0.004). Northerly winds at 9:00 a.m. resulted in increased trapping of coccinellid beetles (Z = 2.369, *p* = 0.018), whereas other bees were trapped less often at northerly winds at 3:00 p.m. (Z = -2.673, *p* = 0.008).

There were only 25 days of rainfall over the observation period and only 5 of these exceeded 5 mm, resulting in no evident relationship between any insect grouping and rainfall. Hours of bright sunshine varied over 34-fold between days (0.4–13.8 h), with an increase in the number of bright sunshine hours significantly associated with a greater occurrence of other bees in the traps (Z = 2.099, *p* = 0.036). No relationship between any of the weather variables tested and the occurrence of either *Phyllotocus* spp. or muscoid flies in traps was evident.

### 3.3. Pollen Carrying Capacity, Inter-Row Movement, and Visitation Frequency to Male-Fertile and Male-Sterile Umbels

**Figure 3:**
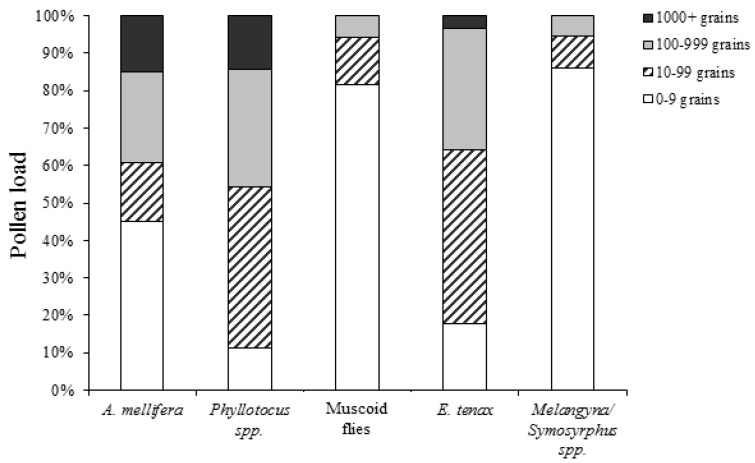
Pollen grains collected from insects in a carrot crop in the first season. Pollen loads are grouped into four categories: 0–9 grains, 10–99 grains, 100–999 grains, and 1000+ grains. Sample size (from left to right) *n =* 133, 35, 243, 56, and 36.

The pollen load carried on insect bodies was not independent of insect groupings (χ^2^ = 181.47, df = 12, *p* < 0.00001; Figure 3). *A. mellifera*, *P. rufipennis*, and *E. tenax* carried more pollen grains on their body than either muscoid flies or *Melangyna*/*Symosyrphus* spp. The majority of the latter two groupings carried nine or fewer pollen grains.

**Figure 4:**
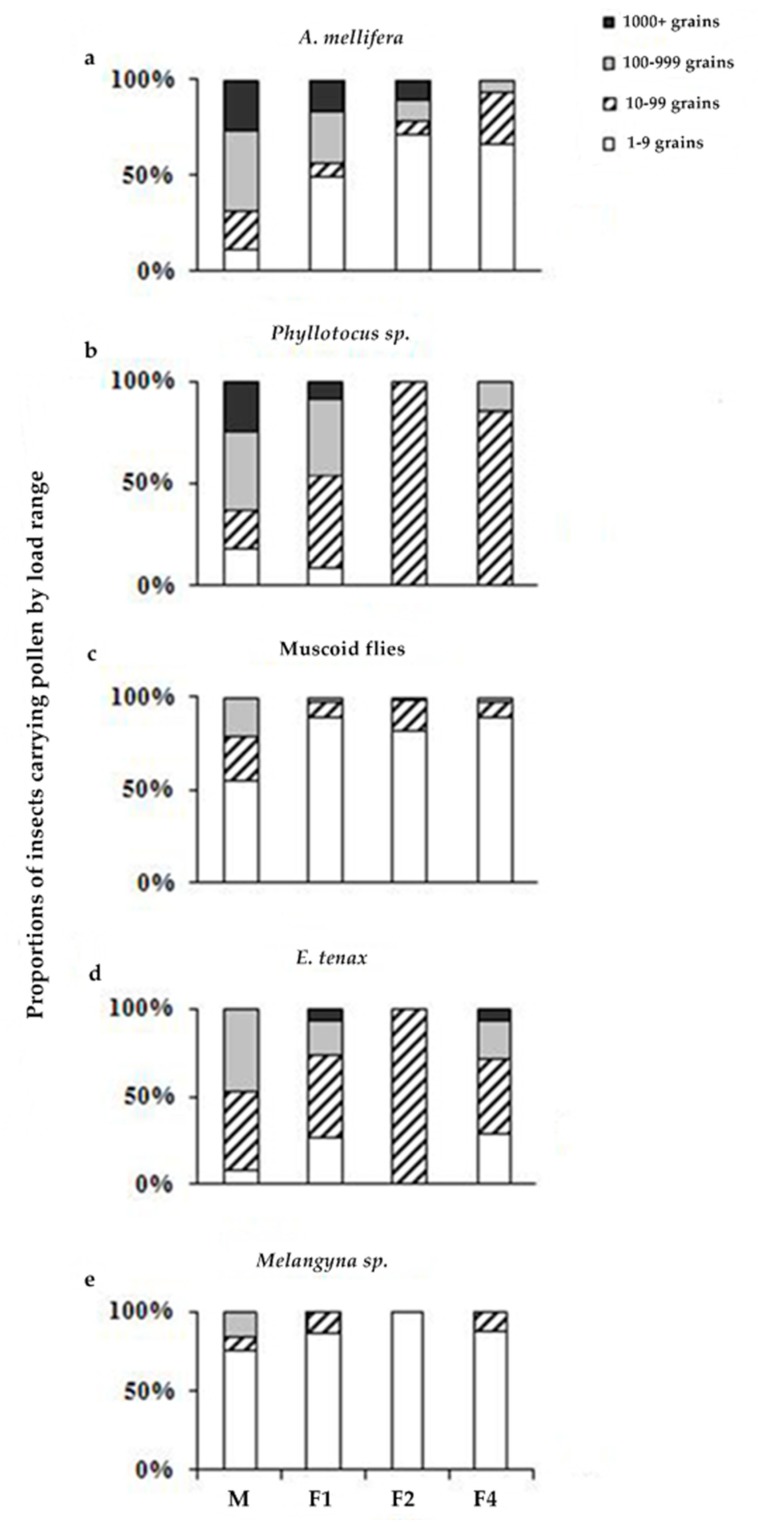
Pollen grains collected from insects in a carrot crop in the first season. M = male-fertile row, F1 = cytoplasmically male-sterile row adjacent to row M, and F2 and F4 rows are respectively two and four rows away from row M. Pollen loads are grouped into four categories: 0–9 grains, 10–99 grains, 100–999 grains, and 1000+ grains. Sample sizes from (**a**–**e**): *n =* 133, 35, 243, 56, and 36 respectively.

The pollen load of the differing insect groupings when collected on the MF row or the first, second, or fourth rows of CMS umbels away from the MF row showed some significant differences (Figure 4). There was a decline in the pollen loads carried by *A. mellifera* the further they were sampled away from the row of MF umbels. For *A. mellifera* and muscoid flies, the pollen load was not independent of collection row, with significantly fewer insects carrying >10 pollen grains furthest away from fertile umbels (honey bees: χ^2^ = 34.76, df = 3, *p* < 0.0001; muscoid flies: (χ^2^ = 25.53, df = 3, *p* < 0.0001, both pooled to two pollen categories: <10 vs. >10 pollen grains on body). This suggests both honey bees and muscoid flies predominantly forage along rows rather than between rows.

However, for *P. rufipennis* and *E. tenax*, pollen load was independent of the row they were collected in, suggesting they forage across rows. For *P. rufipennis,* individuals carrying >100 pollen grains were equally likely to be found on the MF or male-sterile plants (Yates corrected χ^2^ = 2.22, df = 1, *p* = 0.14). *E. tenax* carrying >100 pollen grains were equally likely to be found across the MF row, the first CMS row, and the pooled second and fourth CMS rows (χ^2^ = 2.98, df = 2, *p* = 0.23). The number of *Melangyna/Symosyrphus* spp. carrying high pollen loads was insufficient to statistically test for differences between rows.

**Figure 5:**
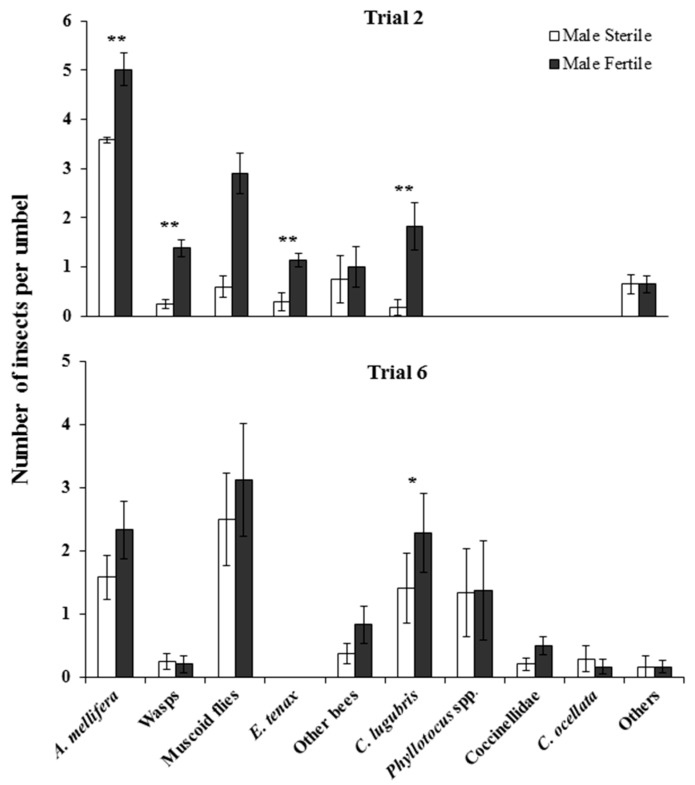
Mean ± SE number of insects observed visiting male-fertile (MF) and cytoplasmically male-sterile carrot (CMS) inflorescences in (**a**) trial 2 and (**b**) trial 6. Trial 2 from left to right *n =* 132, 29, 29, 7, 4, 6, and 17; trial 6 from left to right *n* = 22, 5, 22, 11, 15, 7, 10, 3, and 3. ** Indicates significant difference at *p* < 0.001, * indicates significant difference at *p* < 0.05.

Simultaneously paired observations of insect visitation to MF and CMS umbels by varying insect groupings consistently showed more visitation to MF than CMS umbels in trial 2 and a similar trend in trial 6 (Figure 5). In trial 2, *A. mellifera* (Wilcoxon signed ranks; all *p* < 0.001: z = 3.871), wasps (z = 3.654), and muscoid flies (z = 3.658) all visited MF umbels significantly more often than CMS umbels. Both *C. lugubris* (z = 2.232, *p* = 0.026) and *E. tenax* (z = 2.121, *p* = 0.034) in trial 2 had low visitation rates, but nevertheless had greater visitation to MF umbels than CMS umbels. In trial 6, visitation rates were even lower, and though trends were similar, only *C. lugubris* significantly visited MF umbels more frequently than CMS umbels (z = 2.53, *p* = 0.011).

## 4. Discussion

Insufficient rates of insect-mediated pollination of hybrid carrot seed crops are a key limitation to obtaining optimal hybrid carrot seed yields [3]. In this study, we show that despite the diversity of insect species visiting the crop, few species were found to be frequent, reliable visitors. Of the species that did frequent carrot umbels, only *A. mellifera*, *P. rufipennis*, and *E. tenax* carried high pollen loads. However, of these species, only *A. mellifera* was found to carry adequate pollen, to be in sufficient abundance, and to frequent umbels regularly, implying they were the most reliable pollinators at the sites utilized during this study. Despite this, pollen-load analysis indicated a clear preference by foraging honey bees for the MF parent lines over the CMS lines assessed, thereby limiting their pollination efficiency. These findings concur with those of Erickson and Peterson [31] and Howlett et al. [35]. This low frequency and/or abundance of alternative insect pollinator species, combined with a preferential foraging pattern of *A. mellifera*, may help explain the low seed yields that are often observed in hybrid seed crops including carrot [9,10].

Despite their relatively low densities, numerous insect species were observed throughout this study, many of which are known pollinators of other plants. For example, *Phyllotocus* spp. and *C. lugubris* act as pollinators of *Eucalyptus nitens* in native forests and have been found to carry considerable pollen loads (*Phyllotocus* spp.: up to 13314 grains, and *C. lugubris*: up to 3550 grains per insect) [36]. In native forests, they are particularly prevalent near pasture where *Phyllotocus* larvae feed on roots [37] and cantharid larvae are predatory in the soil [38,39]. Both *P. macleayi* and *P. rufipennis* were observed to swarm in carrot seed crops where they appear to do little or no damage to the carrot flowers. The large numbers of nectar scarabs swarming on carrot inflorescences and soldier beetles with their frequent visits warrant further research into these beetle species. In particular, research should focus on the swarming behavior and the impact of providing suitable soil conditions for their larvae (see Traugott [40] for soldier beetles) near carrot crops to improve pollination levels is warranted.

Generally, insect numbers varied from season to season. In the first season, there were five times more insect visitors than in the two subsequent seasons. The variety of insects differed between seasons and also between the two trials conducted one month apart in December and January of season one. Only *A. mellifera* proved to be reliable visitors, presumably due to the placement of honey bee hives near the crops (with the exception of trial 1). The trapping of insects at two field sites, one with open-pollinated carrots and one with hybrids, in the second season revealed strong similarities between the compositions of insect fauna visiting carrot umbels at both sites. Additionally, although there were occasional disparities in the amplitude of visiting insects between sites (such as the localized influx of *Phyllotocus* beetles and overall significantly greater presence of *C. lugubris* in trial 4), the fluctuation in insect numbers largely coincided between the two sites despite the various carrot types and the insecticide application during trial 3.

Temperature is an important factor influencing insect behavior and affecting the foraging patterns of pollinators [18,27,30,41,42]. *A. mellifera* were trapped more frequently when the daily minimum temperature was higher, most probably because honey bees do not begin to forage until temperatures reach an optimal level [23,30,41]. Similar relationships have been observed between weather conditions and insect mediated pollination of other crops, including bee visitation in apples, kiwi fruit, and raspberry crops [28,43,44]. The group “other beetles” (not including *Phyllotocus* spp. Coccinellidae and *C. lugubris*) was trapped more frequently when the maximum temperature was higher. If pollinator numbers and activity levels increase during periods of higher temperatures, it could be expected that this would result in greater pollination rates at warmer locations. If the increased activity at higher temperatures also leads to an increased foraging range, which in the context of this study would mean increased movement between carrot rows. This may also lead to greater pollination success due to increased movement between MF and CMS umbels. However, it is also expected that the elevated temperatures predicted due to climate change may negatively impact some pollinator species, which may affect wild pollinator foraging in the future [30].

During the observation trials it appeared that the beetles (*P. rufipennis*, *P. macleayi*, and *C. lugubris*) gather on umbels for mating and feeding and use the umbels for shelter in the latter half of the day, whereas muscoid flies, wasps, and bees spend less time on any one umbel than the beetles and move between inflorescences more often. Nectar scarabs (*Phyllotocus* spp.) utilized the umbels to find mates and either arrived there by flight or crawled from one umbel to another. These insects appeared to be foraging on the flowers for nectar or pollen and were prolific during a short one-week period in each of the crops each season. It is also curious that *Phyllotocus* spp. did not remain on the carrot flowers during the full flowering season despite being active throughout the whole summer [45]. Overall, insects visiting umbels for other reasons than foraging for nectar or pollen could still be important pollinators and an extended knowledge of visitation patterns of each species is important for the identification of potential future key pollinators.

There is a worldwide discussion about the value of various pollinators and pollination strategies [32]. The main question has been whether future investments and improvements should be focused on honey bee pollination programs or to explore wild pollinator options. There has been much discussion on the importance of wild pollinators, especially with pests and diseases threatening honey bees [46,47]. There are several examples where diligent management of native pollinators have improved crop yields (see [8]). Although it is important to distinguish between pollen carrying capacity and pollination ability, the latter requiring the deposition of viable pollen on a fertile stigma, we believe that the outcome of our study shows that both honey bees and native pollinators, such as nectar scarabs, should be considered as long-term candidates for hybrid carrot pollination management, as has been proposed elsewhere [14]. In the short to medium term, the strengths of honey bees as pollinators and established beekeeping methods cannot be overlooked. Improving beekeeping practices tailored to crop pollination [48], considering honey bee attraction as a prioritized trait in plant breeding programs, and designing insecticide applications to care for secondary pollinators are some options that could assist in the improvement of pollination.

In conclusion, observations and trapping of insects in hybrid carrot crops reveal that there is a large and diverse assemblage of potential pollinators. However, the irregular arrival and departure of the various insect species within a carrot crop means that most wild pollinators can only be relied upon secondarily. Our findings show honey bees are among the highest pollen carriers and are common flower visitors in carrot. They are also the only species that are managed as a pollinator in Tasmania and, therefore, currently the most reliable pollinator available.

## Figures and Tables

**Table 1:** Details of trials for insect surveys.

Season	Trial	Site *	Date	Total Site Area (m^2^)	Area Used in Trial (m^2^)	Trial Type	Number of Bee Hives	Carrot Type (No. of Cultivars)	Date of Insecticide Spraying (Dominex^®^)
1	1	B	2 December	231	192	Obs	No	CMS (17)	15 January
1	2	SA	3 January	40,000	288	Obs	>1	CMS (1)	Unknown
2	3	SA	3 December–4 January	40,000	300	Trap	>1	Open-pollinated	3 January 17 January 3 February
2	4	UF	3 December–4 January	1100	240	Trap	1	CMS (1)	6 February
2	5	UF	4 January–4 February	1100	690	Obs	1	CMS (6)	6 February
3	6	B	4 December–5 January	1600	260	Obs	1	CMS (2)	None

* B = Bejo seeds; SA = Strath Ayr Turf Systems; UF = University Farm.

## Supplementary Materials

The following are available online at http://www.mdpi.com/2075-4450/9/2/61/s1: Details about site descriptions and crop management procedures, Figures S1–S7, Tables S1–S3.
