# Supplementary material for: Frequent Insect Visitors Are Not Always Pollen Carriers in Hybrid Carrot Pollination
Source: Insects. 2018 Jun 7;9(2):61. doi: 10.3390/insects9020061 (PMC6023358; doi:10.3390/insects9020061)
Supplement: Supplementary file 1 [file insects-09-00061-s001.pdf]

Supplemenary information: Site descriptions

Experiments were conducted at three sites in southern Tasmania during three consecutive summer seasons from 2001/2002 to 2003/2004. Experimental sites were located at

i)	Bejo Seeds Pty. Ltd.,	(42.704° S, 147.445° E)
ii)	StrathAyr Turf Systems Pty. Ltd.	(42.755° S, 147.403° E)
iii)	University Farm, University of Tasmania	(42.797° S, 147.426° E).

All sites were within 30 km of Hobart and within 15 km of each other (Figure).

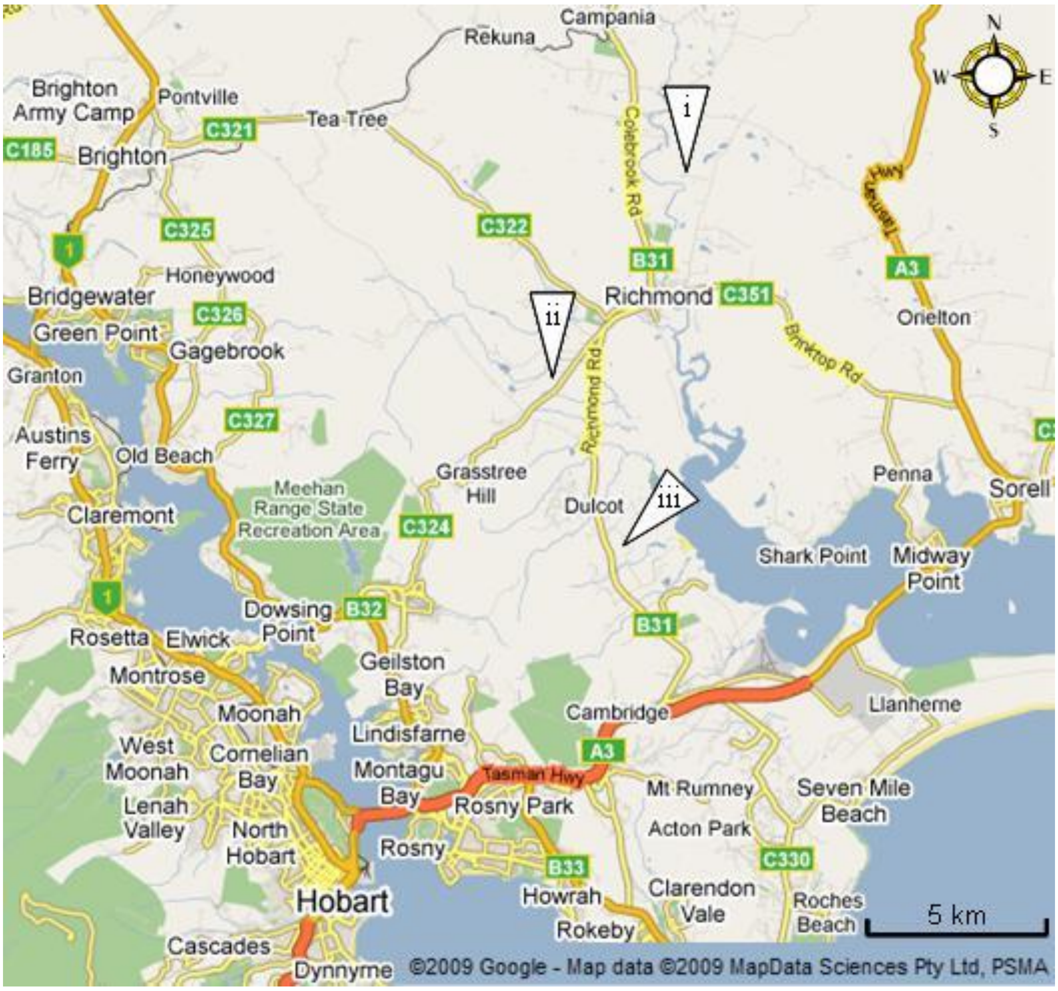

Figure S1. Location of trial sites. - i =Bejo Seeds Pty. Ltd, (42.704°S, 147.445°E), ii=StrathAyr Turf Systems Pty Ltd.(42.755°S, 147.403°E), iii)=University Farm, University of Tasmania (42.797°S, 147.426°E)

Field Trial Layouts

Standard Carrot Planting Layout

The carrots used in this study, unless specified otherwise, were planted in beds 1.2 metres wide. The three rows of carrots were planted lengthwise along the centre of the beds with a distance of 30 cm between each row and a space of 60 cm between the outside rows of carrots in adjacent beds.

<u>Season 1</u>

Field Trial 1 – December 2002 – Site i) Bejo Seeds Pty. Ltd. (192 m^2)

The carrot plants used in this trial were planted for a commercial grow-out trial. These plants were used to test the attractiveness to insects of 17 different cultivars of CMS carrots (BN1, BB1, BN2, PBF1, PN3, PB2, PA1, BN4, BC1, PF1, PN5, PI1, PC2, PBF2, PI2, PK1, and PB3) in a completely randomised design. Carrots were planted in a 19.2 m x 12 m block, which was divided into 24 plots. Seventeen carrot lines were allocated to these plots. Each plot was a 5 m x 1.6 m bed of carrots containing three rows of carrots. The rows of carrots in each bed were 0.3 m distant from each other with a 60 cm space between each of the different carrot lines. Of the additional seven plots, six contained duplicated carrot lines not used in this study and one was empty (Figure). Beds of MF carrot lines were planted at the eastern end of the experimental plot.

Seven field trials were conducted at these three sites over three field seasons. Insect trapping and observations were conducted and umbel trimming was used at some sites to promote lateral flower stem development so that pollinator activity could be observed over an extended carrot growing season. The crop area of these sites and the activities conducted for each trial are detailed in Table S1. Details of these trials can be found in the supplementary materials.

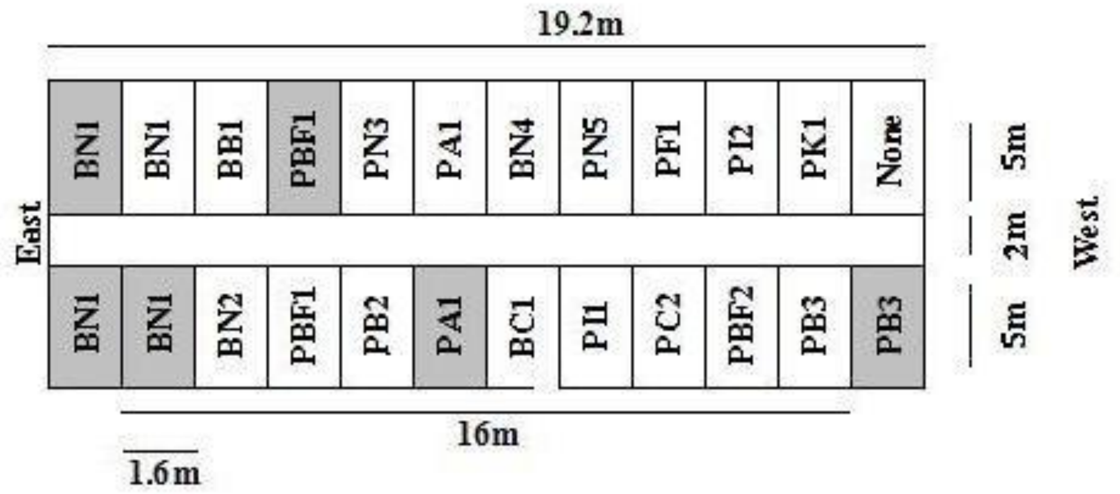

Figure S2. Field Trial 1 – December 2002 - Commercial grow-out trial. Site 1; Bejo Seeds P/L (whole site = 230.4 m^2)

Trial 2 – January 2003 – Site ii) StrathAyr Turf Systems Pty. Ltd. (288 m^2)

Carrots were planted according to the standard carrot planting layout detailed above. The planting layout was consistent with commercial carrot seed crop layout i.e. two beds of MF carrots (type MY1), six beds of CMS carrots (type PN6) then two beds of MF carrots (Type MY1). Only the CMS plants (PN6) were included in this experiment. A 60 m x 4.8 m section of this crop containing six beds of CMS carrots (cultivar PN6) was marked out and divided lengthwise into two blocks. Each of these blocks was then divided into six plots which were 5 m x 4.8 m each. Six different trimming treatments were randomly allocated to the plots within each of the two blocks. Each treatment plot was 1.8 m x 5 m (Figure).

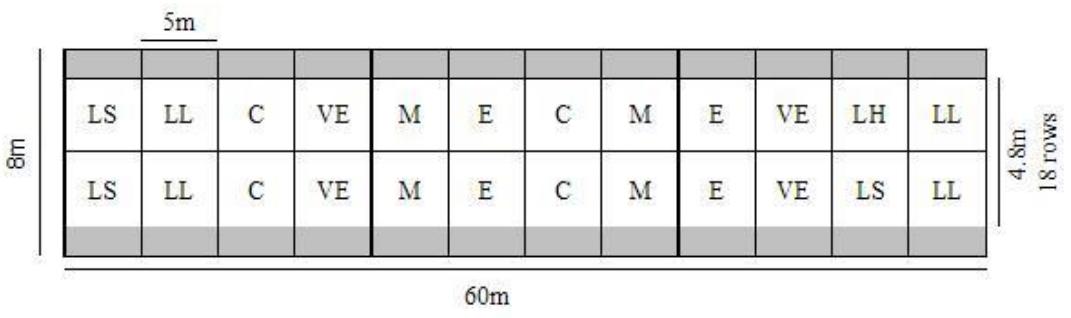

Figure S3. Field Trial 2 – January 2003 - Site ii – StrathAyr 108 m^2 (whole site = 40000 m^2). Treatments; C=Control, VE=Very Early, E=Early, M=Mid, LL=Late Trim – Light, LS=Late Trim – Severe.

All of the carrot trimming treatments were conducted using a line trimmer. The *very early*, *early* and *mid* treatments were trimmed so that all vegetation 50 cm above ground level was removed. The first trimming treatment was conducted when 50 % of the carrots were at an extension of 30 cm or more. Trimming of the *late* trimming treatments were conducted at the same time, just prior to the opening of primary umbels. Carrot plants in the *late-severe* treatment were trimmed to 60 cm above ground level and carrot plants in the *late-light* treatment were trimmed to 75 cm above ground level. Trimming treatments and dates are listed in Table S3.

Table S1. Trimming dates of carrots used in Field Trial 2

Treatment Name	Trimming Date
Control	Not Trimmed
Very Early	14 October 2002
Early	30 October 2002
Mid	13 November 2002
Late - Severe	27 November 2002
Late - Light	27 November 2002

Season 2

Field Trial 3- January 2004 – Site ii) StrathAyr Turf Systems Pty. Ltd.(300 m^2)

This experiment was situated within a commercial crop laid out as in the standard carrot planting layout described above. The carrots in this trial were open-pollinated (MZ1). A random block design was used. The trial was divided into five blocks of six beds of carrots. These blocks were 2.4 m x 25 m. Five treatment plots of 5 m x 2.4 m (9 rows of carrots) were randomly allocated to each block (Figure). Trimming was conducted in accordance with the method laid out for field trial 2. Only treatments control, early, mid, late-severe and late-light were used. Trimming treatments and dates are listed in Table .

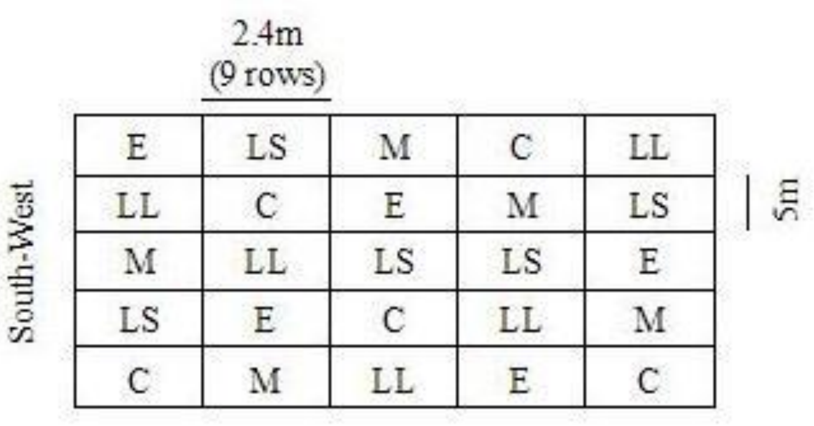

Figure S4. Field Trial 3- January 2004 - Site ii. - StrathAyr. Open pollinated – 300 m^2 (site = 40,000 m^2). Treatments; C=Control, E=Early, M=Mid, LL=Late Trim – Light, LS=Late Trim – Severe**.**

Table S2. Trimming dates of carrots used in field trial 3

Treatment Name	Trimming Date
Control	Not Trimmed
Early	22 October 2003
Mid	12 November 2003
Late - Severe	27 November 2003
Late - Light	27 November 2003

Trial 4 – December 2003/January 2004 – Site iii) University Farm (260 m^2)

The planting layout and carrot cultivar in this trial, MY1 and PN6, were the same as those used in trial 2. Only treatments *control*, *early* and *mid*, *late-severe and late-light* were used. Twenty different treatment plots 2.4 m x 5 m were randomly allocated to a 50 m x 4.8 m block of CMS cultivar PN6. Each treatment plot was 2.4 m x 5 m (Figure). Trimming treatments and dates are listed in Table .

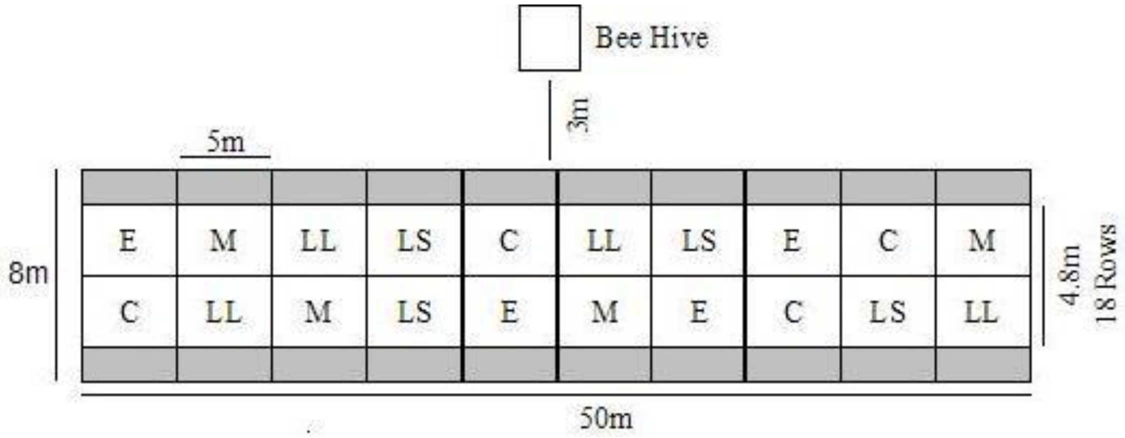

Figure S5. Field Trial 4 – December 2003/January 2004 - Site iii. – Uni Farm 400 m^2 (whole site = 1100 m^2). Treatments; C=Control, E=Early, M=Mid, LL=Late Trim – Light, LS=Late Trim – Severe.

Table S3. Trimming dates of carrots used in field trial 4

Treatment Name	Trimming Date
Control	Not Trimmed
Early	15 November 2003
Mid	1 December 2003
Late - Severe	23 December 2003
Late - Light	23 December 2003

Trial 5 – January 2004- – Site iii) University Farm (690 m^2)

Five blocks of 10 m x 0.8 m were planted with a single bed (3 rows lengthwise) of each of six carrot cultivars (PBF1, PC2, PN3, PA1, PB3 & PF1) in a random block design. A single bed (3 rows) of one MF cultivar (MX1) was planted in between each of the experimental CMS cultivars to separate the plots and to stimulate pollinating insects, as is the practice in commercial crops. A 4 m gap was left in between each cultivar (Figure). One block of cultivar PA1 failed to germinate leaving five blocks of each of the other cultivars and four blocks of PA1.

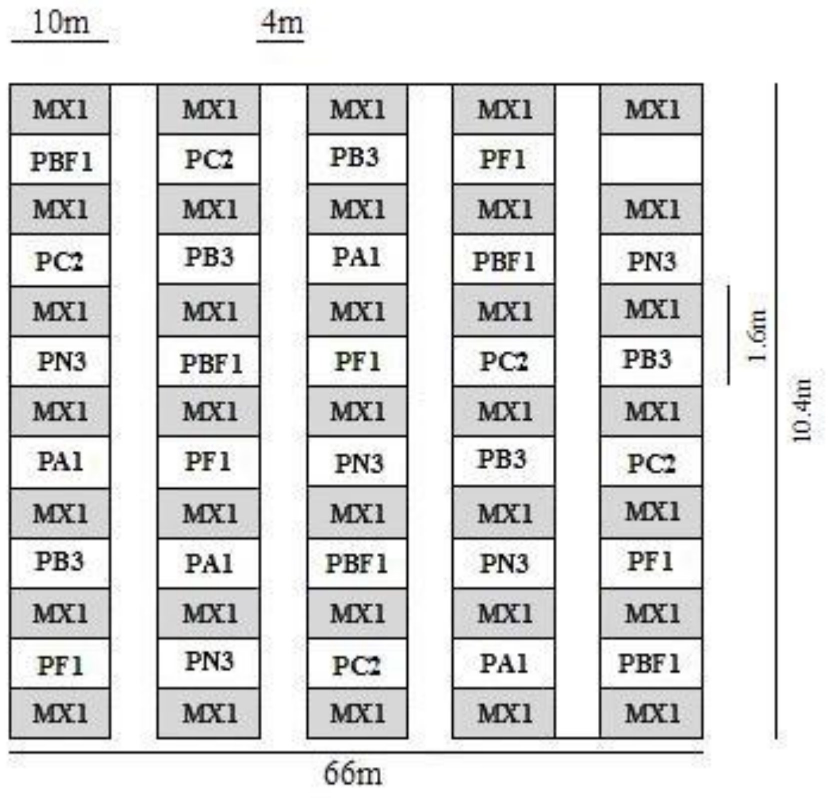

Figure S6. Field Trial 5 – January 2004 - Site iii – University Farm 690 m^2 (whole site = 1100 m^2)

Season 3

Trial 6 – December 2004/January 2005 – Site i) Bejo Seeds Pty. Ltd. (260 m^2)

This trial was a randomised block design. Eight blocks were divided into two plots of 2.4 x 5 m. Each plot was planted with one bed of an MF carrot (MX1) and then two beds of either carrot cultivar PF1 or PBF1. Each block contained a plot of PF1 and a plot of PBF1 (Figure S7).

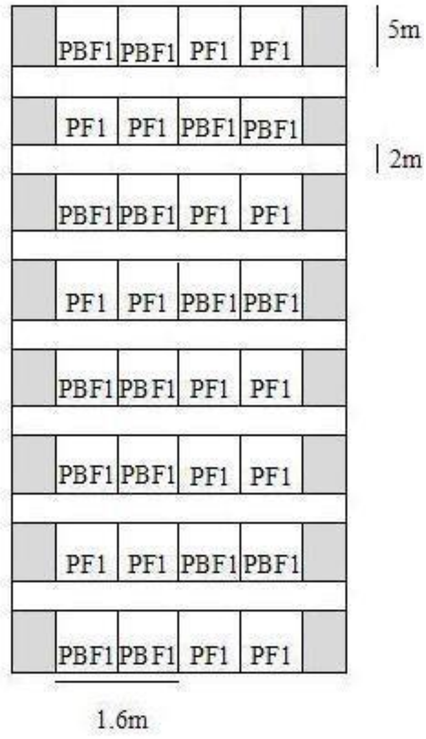

Figure S7. Trial 6 – December 2004/January 2005 – Site i – Bejo study area = 260 m^2 whole site =1600 m^2)
